# Alcohol Use Amongst Rural Adolescents and Young Adults: A Brief Review of the Literature

**DOI:** 10.1177/00332941241251460

**Published:** 2024-04-26

**Authors:** Jessica Saalfield, Bethany Haag

**Affiliations:** Deparatement of Psychology, 52531 Penn State Schuylkill, Schuylkill Haven, PA, USA; Deparatement of Psychology, 52531 Penn State Schuylkill, Schuylkill Haven, PA, USA; Department of Biobehavioral Health, Penn State, University Park, PA, USA

**Keywords:** alcohol, rural, young adult, adolescence, geography

## Abstract

The sociodevelopmental periods of adolescence and young adulthood are rife with alcohol use. However, much of the literature demonstrating this comes from ‘traditional’ settings and college campuses (i.e., large suburban/urban campuses, or those containing their own infrastructure). Alcohol culture in rural areas has largely been understudied, which may be problematic given the unique stressors they face (e.g., economic hardship, lack of social activities, healthcare inequality). There has also been difficulty both within and across fields classifying rural versus urban geographical locations; no distinct system used broadly, making ittrea difficult to generalize and accurately collect data. The geographic categorizations are often viewed as homogenous identifiers; however, diversity occurs both within and outside of these classification systems. It appears that rurality may be a risk factor for increased drinking both earlier and later in life, but the research has failed to extend to the formative college years. This short review has two main focuses: attempting to disentangle the definition of rurality and reviewing the literature regarding alcohol use in rural areas, with a specific focus on adolescents and young adults. Identifying the mechanisms responsible for substance use in rural areas is a crucial component of prevention and treatment programs.

## Introduction

For decades, rural communities have struggled with decreasing resources, populations that are harder to access, and economic disadvantage, among other privations. The opioid crisis, that is now over two decades old, magnified the catastrophic changes in rural America that have led to shorter life spans, greater poverty, and increased drug use (Compton et al., 2021; Ghertner & Groves, 2018; Muennig et al., 2018), including increased rates of episodic binge and extreme drinking (T. F. Borders & Booth, 2007). These communities also lack access to substance treatment programs and other mental health services (Friesen et al., 2023; Jiang et al., 2008; Snell-Rood et al., 2022). Despite the risk environment (i.e., physical spaces, stigma, health concerns, oppression, etc., see Rhodes, 2009) that works to shape the harms attributable to rural communities, these areas have been under-represented in alcohol research. This short review will focus on the limited literature regarding alcohol use in rural areas, with a specific focus on the adolescent and young adult epoch, while concomitantly highlighting areas where more research is needed.

## Rural and Urban (Defined)

A complication with examining alcohol use in reference to geography is the diversity within the conceptualization of rurality. There is no distinct classification used, with a myriad of definitions of rural in use by both research and governing bodies (e.g., see Bennett et al., 2019; Friesen et al., 2022; Showalter, 2020). Briefly, within the United States some studies use the Rural-Urban Continuum Codes set by the United States Department of Agriculture (e.g., Derefinko et al., 2018; Lambert et al., 2008; Warren et al., 2015) while others use Metropolitan-Micropolitan Statistical Areas (e.g., Booth et al., 2000; Patrick et al., 2013). In Australia, most studies define rurality according to the Australian Standard Geography Standard remoteness structure (Azar et al., 2016; Rowe et al., 2012; Roxburgh et al., 2013) or the Accessibility-Remoteness Index of Australia (e.g., Burns et al., 2011). On the contrary, additional studies utilize qualitive self-reported data, such as postal codes (McKay & Cole, 2017) or a variety of data compiled into a unique scale (Schultz & Neighbors, 2007). Other studies focus solely on population size (e.g., Jiang et al., 2008; Shears et al., 2006), but they often utilize different limits to delineate geographic designations, complicating matters even further. Lastly, geographic designation is often represented in multiple ways, including as a dichotomous variable (i.e., rural – urban) or as a continuous variable with multiple categories. Surprisingly, a large number of studies claiming to compare alcohol use and/or alcohol-related harms across geographic regions failed to provide a definition of rural within the study (Friesen et al., 2022).

Rurality can also be defined by cultural characteristics, including topics such as acceptance of substance use, religiosity, and availability of substances (Derefinko et al., 2018). For example, Warren et al. (2015) used county-level definitions of rurality based on the codes developed by the Health Resources and Services Administration’s rurality designations. McKay and Cole (2017) also suggest that the various schemes across geographic designations vary across the United States while also extending into other countries.

Studies of rural areas tend to focus on the population size and geographical parameters of the area while failing to consider traffic patterns. High travelled roadways often intersect with rural areas and have the potential to allow for increased drug trade. Sociocultural factors of living in a rural area include a relaxed attitude towards alcohol and higher rates of adult substance use (Gale et al., 2012). Rural areas also have a lack of recreational opportunities for a span of all ages (McKay & Cole, 2017). Research from Borders and colleagues (2020) demonstrates that not only is alcohol the most used substance across varied geographical settings, but there are also higher rates of binge drinking noted in non-metropolitan areas (T. Borders et al., 2020). Overall, it is not surprising that there are mixed findings assessing substance use in rural areas given the variety of geographic classifications and definitions used. The scientific field should attempt to reach a consensus regarding what qualifies as rural to make research findings more generalizable and translatable.

## Hardships

Rural areas are often characterized by decreased access to healthcare and limited economic opportunities which are often compounded by lengthy distances to large towns with proper resources (Lenardson et al., 2012). The limited number of treatment programs available for rural populations are frequently lacking as a result of cultural differences and stigmas against mental disorders and substance use (Broffman et al., 2017; see Snell-Rood et al., 2022 for a review). Individuals in rural areas tend to experience financial and economic difficulties, alcohol may be seen as an easy way to self-medicate leading to increased amounts of hazardous alcohol use. Rural areas are regularly under-policed and have smaller budgets/fewer resources for substance use may be directed elsewhere (Weisner et al., 2020). Due to other stressors of location and cultural attitudes, rural geographic regions are linked to higher rates of driving under the influence (Friesen et al., 2022), which is concerning noting the policing issues in these areas.

## General Alcohol Use

Alcohol is the most widely used substance among a variety of demographics and cost the United States approximately $249 billion on concerns including legal, healthcare, and workplace loss (Sacks et al., 2015). Individuals with alcohol use disorder experience an elevated risk of co-occurring/concomitant of physical and psychological disorders (Jeanblanc, 2015). Alcohol use differs between countries, cultures, geographical regions, and personal demographics, therefore varying the research outcomes done within each area (Friesen et al., 2022). Alcohol use experimentation tends to begin during the adolescent stage of initiation for drug and alcohol use. This introduction to substance use pairs with potentially risky behaviors during middle and high school.

## High School Alcohol Use

The adolescent period is marked by individuals experimenting with substance use; early experimentation of alcohol use can cause AUD or substance use-related issues in the future (e.g., Maggs et al., 2022 for a review). The rising presence of social media exposes adolescents to pro-substance use among their peers, often encouraging adolescents to partake in the activity (Patton et al., 2016). As adolescents begin sexual experimentation and hold increased sexual awareness, alcohol use during this time can contribute to risky behaviors (Garcia et al., 2019). The addition of alcohol use to sexual relationships in adolescence means there is cognitive impairment during sexual encounters (Garcia et al., 2019), further exacerbating the negative effects of the increased risk taking associated with adolescence. The parent-child relationship also affects the way adolescents approach the subject of alcohol use; parenting styles that did not discourage underage drinking resulted in a higher likelihood of increased rates of alcohol use (Gale et al., 2012). Adolescents perceive an easy access to alcohol, namely in rural areas (Warren et al., 2015). Regardless, literature has failed to immerse itself in adolescent alcohol use in rural areas, leading to a dearth of knowledge on the topic, subsequently leading to ineffective prevention and intervention policies.

### Effect of Rurality

Adolescents in rural communities are more likely to engage in hazardous alcohol use, including meeting criteria for alcohol use disorder (Friesen et al., 2022). Furthermore, adolescents in rural areas not only drink alcohol at higher rates than those in urban areas (Dixon & Chartier, 2016; Donath et al., 2011) but engage in numerous risky behaviors because of alcohol intoxication (e.g., higher rates of drunk driving) and also begin consuming alcohol at an earlier age (Chan et al., 2016; Coomber et al., 2011; Gale et al., 2012; Lambert et al., 2008; Martin et al., 2019). Early age of alcohol initiation, in conjunction with a high volume of consumption, are indicators of developing substance use problems in the future (e.g., see Dawson et al., 2008; DeWit et al., 2000; Friesen et al., 2022). Adolescents in rural areas perceive binge drinking as less risky than adolescents in urban areas (Saalfield, Haag, and Ryerson, submitted), which may be contributing to the higher rates of extreme binge drinking (+15 drinks per occasion) noted amongst rural teens (Patrick et al., 2013; Livingston et al., 2008; Obradors-Rial et al., 2020; Saalfield et al., submitted). There is also limited evidence on the effectiveness of intervention programs aimed at addressing this increased alcohol consumption in rural youth (Cibich et al., 2023).

The cultural attitudes associated with alcohol use in rural areas contribute to high school adolescents reporting higher rates of substance use (Lambert et al., 2008), namely alcohol (Friesen et al., 2022; Schultz & Neighbors, 2007) when compared to their urban counterparts. This occurrence is partly due to the perceived ease of access by high school students in which “legal” substances are believed to be easier to obtain (Warren et al., 2015). Though the exact reason for the ease of access is unknown, it is hypothesized high school adolescents living in a rural area are more subjected to family members engaging in substance use (Warren et al., 2015; Gale et al., 2012).

Additionally, structural and cultural aspects of rural living, including increased acceptance and increased isolation, may make rural adolescents more vulnerable to increased substance use (Chan et al., 2016; Gale et al., 2012; Sarvela et al., 1990; Thrash & Warner, 2016). Together, the perception of access, familial influence, and geographic isolation with minimal opportunities for recreational activities may be significant contributors to higher rates of alcohol use among high school adolescents.

## Alcohol Use Amongst Young Adults (College and non-college Patterns)

The transition between high school to college is often characterized by a newfound sense of freedom and lifestyle changes. In the United States, the rates of substance use peak between ages 18 and 25 years (Johnston et al., 2015). College is a time of increased risky behavior, including high rates of binge and heavy drinking. College students consume alcohol for a variety of reasons (e.g., see Merrill & Carey, 2016 for a review), including drinking to increase positive affect (Magid et al., 2007; Merrill & Read, 2010) and drinking to cope with stress (Jones et al., 2014; Merrill & Read, 2010).

College-typical patterns of alcohol use are well-characterized; some of the social determinants related to alcohol use include factors such as Greek life, athletic status, campus norms, etc. (e.g., see Merrill and Carey, 2016). College is a time where individuals are under increased amounts of stress from factors of coursework, finances, and living situation. Coping mechanism predictors of alcohol behavior include impulsive personality traits, alcohol expectancies, and membership in fraternities and sororities (Sher & Rutledge, 2007). Though it must be noted individuals who choose not to engage in drinking behaviors have other issues to consider; Schultz and Neighbors (2007) suggest students who are deviant from drinking norms of college feel alienated from their peers.

Individuals who do not attend college differ from their college-attending counterparts; this may be due, at least in part, to the college environments providing the opportunity for increased and excessive alcohol use. Reviews of the literature have suggested differences in binge-drinking drinking rates amount college versus non-college students (Carter et al., 2010; Merrill & Carey, 2016), with most data demonstrating higher rates of alcohol use amongst college students (Patrick & Terry-McElrath, 2017; Quinn & Fromme, 2011). However, some studies indicate that both groups consume alcohol and similarly heavy rates (Chen et al., 2004; Linden-Carmichael & Lanza, 2018). Regardless of student status, the young adult period is rife with risk for unhealthy alcohol use.

The current literature of alcohol use on college campuses is conducted on large-scale universities. These studies are not fully representative of all colleges in the United States. As mentioned, there is a lack of translation among data which leads to a lack of a generally accepted consensus that applies to all universities. This lack of translation affects how data is interpreted by researchers in the field. This area of substance use research is lacking across multiple levels.

### Effect of Rurality

The research that has been conducted on rates of alcohol use on college campuses is largely focused on more suburban settings. Colleges in more populated areas or that have sizable student bodies have easier access to Greek lifestyle, bars, and other recreational activities that involve alcohol, whereas these opportunities are not often found on small and/or rural college campuses. Rural campuses are characterized by not only size, but the attitude held by residents towards alcohol consumption. These beliefs allow for a cultural acceptance of alcohol in the area.

In terms of alcohol use on rural college campuses, not much is known regarding the effect of rurality as most of the effects of geography are centered around middle or high school student use. Attending a rural colleges has been demonstrated to be a predictor or alcohol use and binge drinking (Haardörfer et al., 2021). College students originating from rural areas consume less alcohol than their urban counterparts as freshman, but soon move to match the rates by their junior year (Derefinko et al., 2018). Yet it has also been suggested students with a rural background drank more frequently and in higher quantities when compared to students with an urban background (Schultz & Neighbors, 2007). Discrepancies in the literature can again be a result of differences between the classifications of rural and urban. There is also a noted sex difference, with female-use decreasing over time and male-use increasing, namely in the rural group (Derefinko et al., 2018). Fromme and colleagues determined that college students that attended rural high schools were at an elevated risk for heavy drinking during their first year in college (Fromme et al., 2008). They also noted that the rates of driving while under the influence tend to decrease once an individual from rural area attends college, as there is more freedom to drink at their own place of residence (Fromme et al., 2008). Research from across the globe has also demonstrated that adolescents and young adults living in rural areas are not only more likely to engage in risky-level drinking but also are at an increased risk of experiencing alcohol-related harms than individuals from more urban areas (Coomber et al., 2011; Miller et al., 2010). Overall, the paucity of research in this area is troublesome given the high rates of problematic alcohol use noted amongst college students and young adults.

## Why Does This Matter?

The previously discussed ease of access of illicit alcohol in combination with the increased alcohol use amongst rural teens and young adults is problematic. These findings have still not been explored in rural college students despite an overlap of features including age, prevalence/availability of substances, and lack of social activities. What little exists is mixed. One finding demonstrated that individuals who attend college coming from a rural area were less likely to use alcohol as a first-year student, but eventually matched the rates of alcohol consumption by their junior year when compared to their urban counterparts (Derefinko et al., 2018), while another suggested that rural students were at elevated risk for elevated drinking in college (Fromme et al., 2008). However, these studies don’t consider the geographic characteristics of the college campus, and the findings may be unable to be extended to other college campuses.

Friesen et al. (2022) cited fifty-five types of risk factors of adults living in a rural community. These factors include smoking, proximity to family members who drink alcohol, childhood trauma and abuse, and a lack of religious involvement. It is unclear how these risks are moderating the increased alcohol intake noted in rural adolescents and young adults. Though living in a rural area is characterized by risk factors of future substance use, some studies suggest rurality serves as a protective factor, though what mechanisms of protection are unknown (Derefinko et al., 2018), lending more support for continued research efforts in this area.

The lack of data on this topic is problematic for many reasons. Having proper data is important in all facets of substance use, from prevention to invention, and in creating effective educational material for individuals in diverse geographic areas. Comparison of substance use programs are not valid when referencing rural areas of geographic populations (Lenardson et al., 2012) making research in this area even more important.

The data of drinking patterns on rural adolescents and young adults is largely understudied. Much of the extant literature is not recent, and data examining drinking among these age groups do not factors in geographical characteristics, threatening the translatability of the data. The mechanisms underlying the differences in alcohol use and behaviors across geography are not well understood and more than likely are multifaceted and complicated. For example, neighborhood and/or cultural characteristics, genetics, socioeconomic status and race may all be factors worth consideration (Chockalingam et al., 2013; Dixon & Chartier, 2016; Donath et al., 2012; Fagan et al., 2015; Jackson et al., 2016; Ma et al., 2018; Martin et al., 2019; Reitsma et al., 2017; Slutske et al., 2016). In all, the limited research is imploring the scientific community to continue studying the effects of geography on substance use in younger populations.

## Data Availability

Data sharing not applicable to this article as no datasets were generated or analyzed during the current study.
